# From Biological Source to Energy Harvesting Device: Surface Protective Ionic Liquid Coatings for Electrical Performance Enhancement of Wood-Based Electronics

**DOI:** 10.3390/molecules28196758

**Published:** 2023-09-22

**Authors:** Gulnur Zharkenova, Emre Arkan, Mesude Zeliha Arkan, Joanna Feder-Kubis, Janusz Koperski, Turlybek Mussabayev, Mirosław Chorążewski

**Affiliations:** 1 Institute of Chemistry, University of Silesia in Katowice, Szkolna 9, 40-006 Katowice, Poland; flower_0525@mail.ru (G.Z.); mesude-zeliha.arkan@us.edu.pl (M.Z.A.); 2 Department of Civil Engineering, L.N. Gumilyov Eurasian National University, Astana 010008, Kazakhstan; eti.enu@mail.ru; 3 Faculty of Chemistry, Wrocław University of Science and Technology, Wybrzeże Wyspiańskiego 27, 50–370 Wrocław, Poland; joanna.feder-kubis@pwr.edu.pl; 4 Department of Inorganic Chemistry, Technische Universität Dresden, 01069 Dresden, Germany; 5 Institute of Physics, University of Silesia in Katowice, St 75 Pułku Piechoty 1, 41–500 Chorzów, Poland; janusz.koperski@us.edu.pl

**Keywords:** renewable natural resource, (−)-menthol, saccharinate-based ionic liquid, wood-based TENG, smart floor

## Abstract

This study explores task-specific ionic liquids (TSILs) in smart floor systems, highlighting their strong electrical rectification abilities and previously established wood preservative properties. Two types of TSILs, featuring a “sweet” anion and a terpene-based cation, were used to treat selected wood samples, allowing for a comparison of their physical and electrical performance with untreated and commercially treated counterparts. Drop shape analysis and scanning electron microscopy were employed to evaluate the surface treatment before and after coating. Near-IR was used to confirm the presence of a surface modifier, and thermogravimetric analysis (TGA) was utilized to assess the thermal features of the treated samples. The different surface treatments resulted in varied triboelectric nanogenerator (TENG) parameters, with the molecular structure and size of the side chains being the key determining factors. The best results were achieved with TSILs, with the instantaneous voltage increasing by approximately five times and the highest voltage reaching 300 V under enhanced loading. This work provides fresh insights into the potential application spectrum of TSILs and opens up new avenues for directly utilizing tested ionic compounds in construction systems.

## 1. Introduction

In the new global economy, biodegradable and eco-friendly materials play a crucial role in the development of renewable industrial processes and are regarded as powerful platforms for sustainable futures [1]. A good example is wood-based materials, which are lightweight, inexpensive, and easy to process, with the added benefit of being recyclable [2,3,4]. Wood is also structurally flexible, has a porous texture, and possesses the benefits of high mechanical impact resistance, low density, a high surface area, and a high aspect ratio [2,5]. Fortunately, the surface treatment of wood enhances its durability against environmental factors, such as fire and climate, and improves its functionality, making it suitable for use in smart home systems [6,7,8,9]. Therefore, exploiting this potential for energy harvesting devices, such as triboelectric nanogenerators (TENGs), is of interest [2,10,11,12,13]. Hao et al. developed TENGs for energy harvesting and motion sensing [10]. Likewise, He et al. fabricated TENGs to harvest energy and sense specific motions, such as walking, running, and jumping [14]. Bang et al. used typical silane-based structures to create tribopositive and tribonegative wood layers [2]. Furthermore, the use of new biocompatible materials as surface modifiers could be an essential next step in improving the effectiveness of smart floor systems.

Most of the literature on TENGs has focused on design, circuit modeling, and improving potential applications [15,16,17,18]. For example, contact area enhancement of triboelectric layers through physical treatment has been explored using various approaches [19,20,21]. However, the molecular characteristics of the applied materials greatly influence surface charge accumulation and, therefore, the triboelectric effect. Using novel and biodegradable materials to enhance the triboelectric effect is relatively uncommon [22,23,24]. It is, therefore, evident that the integration of biobased molecules into wood-based TENG systems could be an effective step in enhancing the device’s performance.

Recent evidence in the literature highlights the benefits of small organic molecules, including their ease of production, well-defined molecular weight, simple purification, exceptional reproducibility, and adjustable molecular structure [25,26]. In this sense, task-specific ionic liquids (TSILs) [27] are promising compounds with desirable properties and reactivities in many fields. TSILs are ionic liquids (ILs) in which the functional group is covalently tethered to the cation or/and anion of the molecule. The characteristics of these salts can be influenced by the cation–anion combination, the nature of the introduced functional group, and its position in the molecule’s structure. TSILs possess favorable ionic conductivity and high thermal and electrochemical stability [28,29,30,31,32,33]. They are suitable for chemical processes under conventional conditions due to their nonflammable and less volatile features, which prevent atmospheric contamination [34,35,36,37]. The functional groups incorporated into the structure of TSILs also provide biological specificity, making them highly recognized in biotechnology [33,38,39] and pharmacology [40,41]. These benefits position them as environmentally friendly chemicals, offering advantages over commercial chemicals in surface-coating applications. Furthermore, the molecular structure of TSILs is particularly well suited for applications in energetic materials, with their structures forming ideal ionic pairs, making them attractive components in electronic devices, such as redox couples in dye-sensitized solar cells and high-energy density providers in lithium battery technology [42,43]. Their unique configurations when paired with certain metals and lanthanides, such as Fe^3+^ and Dy^3+^, give them paramagnetic characteristics and luminescence behavior, rendering them valuable for future advanced computer and display technology [44,45,46]. Moreover, the high nitrogen content in the imidazolium cation of their chemical structure allows the formation of carbamate upon interaction with CO_2_, facilitating their usage in CO_2_ gas sensor studies [47,48]. This nitrogen content is particularly considered for applications in explosives and their safe handling [49]. While TSILs have demonstrated potential in various applications, from pharmaceuticals to organic electronics and surface-coating agents, further comparative studies are needed to investigate their multifunctional abilities as surface-coating agents for wood-based materials, especially in enhancing the thermal and electrical properties of smart floor systems [50,51,52,53]. Therefore, any investigation focusing on this research gap is expected to significantly contribute to the literature.

This work aims to use TSILs as unique surface modifiers to evaluate the physical and electrical properties of different wood samples, specifically spruce, larch, and pine, which are commonly used in the construction sector. Studies on the potential application of TSILs in the wood industry are scarce but promising and have primarily focused on their wood preservative properties [34,54].

Our previous work [33] demonstrated that quaternary imidazolium salts with multiple functional groups could be successfully applied in the wood industry. Apart from promising results for wood preservation, tested salts were found to have low aggressiveness against carbon steel, even lower than commercially used patterns. Thus, we decided to further investigate these surfactants belonging to TSILs. We hypothesized that utilizing them in smart floor systems could serve as a model approach to influence further relevant research and expand their potential usage in the future market. For this purpose, two types of TSILs and a commercial coating were employed to treat wood samples, and their pros and cons were compared. The electrical measurements were based on fabricating wood-based TENG devices using properly sliced wood and coating materials. Surface-coated woods were used as tribopositive layers in all device measurements, and the device’s performance was measured under different loads. The experimental work presented here provides important insights by illustrating the application of TSIL-based coatings as a performance booster in smart floor systems and their effectiveness as wood surface protection agents.

## 2. Results and Discussion

**Figure 1:**
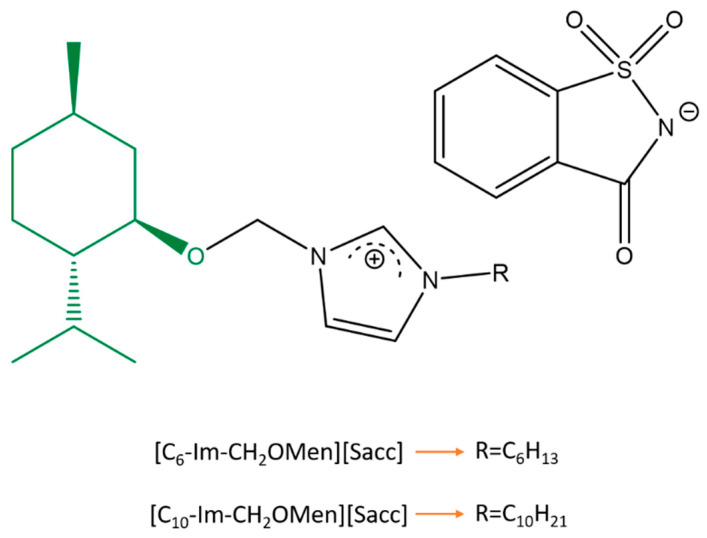
Molecular structure of TSILs used in this study.

In the present work, two TSILs possessing two functional groups, including (–)-menthol as the cation and saccharinate as the counterion, which varied in alkyl chain length (hexyl and decyl substituent), were used to modify the selected wood surfaces (Figure 1). Since the effectiveness of tested quaternary imidazolium salts as anti-fungal agents in wood preservation has been proven in a previous study [33], further evaluation to test the thermal performance and electrical behaviors of TSILs is needed to support the application of this powerful platform in a wide range of technologies, such as smart floors. To this end, three types of wood—spruce, larch, and pine—have been selected due to their extensive exploitation for tough uses in industry and civil engineering construction. To evaluate the synergism of the obtained parameters and to create a correlation between them, a comparative screening experiment was performed using commercial coatings. Moreover, wood is mainly used outside and subjected to atmospheric conditions in real life; therefore, its thermophysical properties before and after the coating are specifically important for long-term performance. PTFE tape and each prepared wood sample, both coated and uncoated, were chosen as opposite layers to enhance the triboelectric power of the device. PTFE tape is known for its tribonegative nature, while wood is tribopositive. Additionally, these materials are cost-effective and lightweight, and have a large specific surface area.

**Figure 2:**
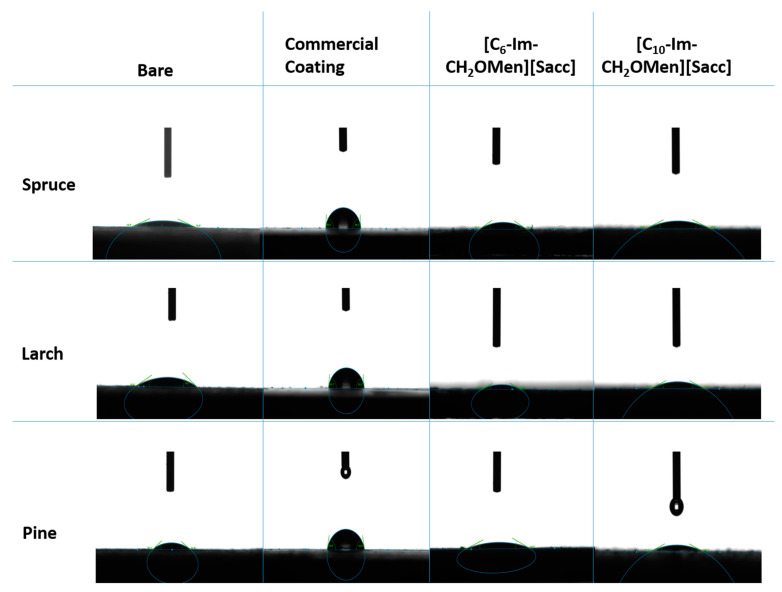
Changes in water contact angles with respect to different surfaces.

Analyses were performed to examine the wetting behavior of the wood surface before and after coating to evaluate the coating effect of molecules on the surfaces. To achieve this, sessile water droplet measurements were conducted on the surfaces at room temperature, and data were collected after the water droplet completed its spread. Contact angle measurements were collected for all wood coupons, and a similar trend was found for contact angles before and after IL coatings. However, GT-9 had the highest contact angle among the group of bare woods, whereas GT-4 showed the highest contact angle for IL-coated samples. It is well known that the polar nature of water, having a partially negative and partially positive structure, plays a decisive role in the interaction of bulk water with any substance or surface. Therefore, grafting the wood surface with polar organic moieties or charged molecules will yield ideal wettability [55]. This is why IL coatings on all woods show wettable features, making them a suitable surface modification platform. These findings are consistent with previous studies that indicate TSILs penetrate the wood to some extent rather than merely staying on the surface [34]. Moreover, this wettability feature of TSILs can be regarded as superior to their hydrophobic counterparts in terms of triboelectrification. Thanks to the hydrophilic surface formed by these ILs, water molecules are expected to participate in triboelectrification, thereby enhancing triboelectric surface charge generation and increasing the output performance [56,57]. Conversely, non-polar entities, such as homogeneous hydrocarbon chains, exhibit more hydrophobic properties due to the free energy transfer from the aqueous phase to the hydrocarbon structure [55,58,59,60]. As shown in Table 1 and Figure 2, double-layered commercial coatings have increased the hydrophobicity of the surface due to their mixture of hydrocarbon and aromatic structures. Additionally, it was observed that, unlike TSILs, the bottom coat of commercial materials did not penetrate as deeply and instead created a solid layer after applying top coatings. The relationship between surface coatings and contact angle values confirms that the type of grafting material significantly impacts surface behaviors and is also expected to affect related physical, thermal, and electrical properties.

**Figure 3:**
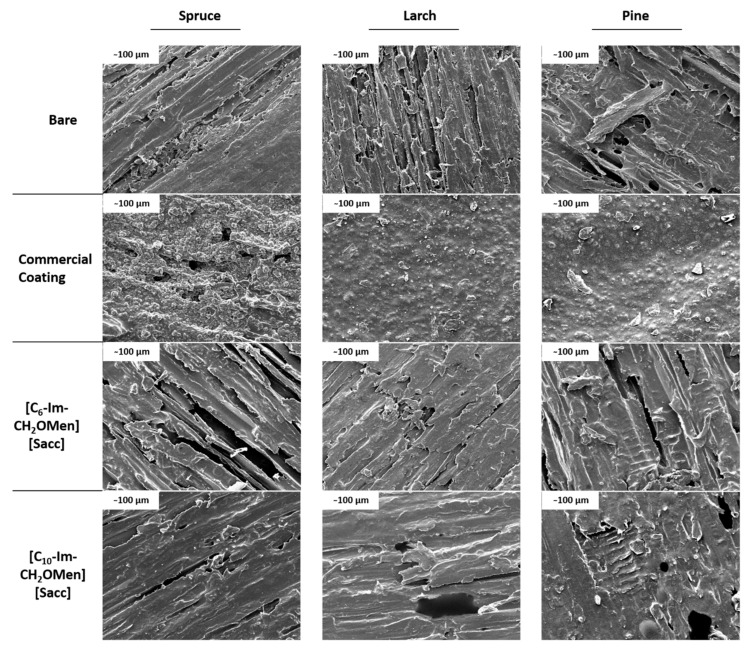
SEM images of wood surfaces used in TENG fabrication.

The SEM technique was employed to image the samples before and after coating to determine how the coating type regulates the material surface, which has a crucial impact on crafting the material for engineering applications. The images in Figure 3 clearly show that wood structures naturally have a crater-type surface arrangement. However, commercial coatings have filled these spaces to a significant extent and even resulted in sedimentation on some parts of the surface due to polymerization after top-layer coating. This sedimentation could be another reason for the high contact angle of commercial coatings. In contrast, TSILs did not form any external layer on the wood surfaces, and there was no significant change in surface morphology before and after ionic compound coating. This further supports the finding that TSILs penetrated the wood matrix by replacing moisture in the gaps.

In a previous study by Kubis et al., the presence of quaternary imidazolium salts on the wood surface was evaluated using ATR-IR [34]. In the current study, we gained further insight into the surface coverage of wood samples through near-IR spectroscopy, and the resulting spectra are shown in Figures S1–S3. Since these TSILs penetrated the wood structure, peaks in the spectra of bare and coated woods only differed in small shifts, indicating the presence of some specific groups related to ILs. Conversely, commercial coatings created specific layers on the top of wood surfaces rather than penetrating the wood. Consequently, the absorption intensity of the peaks increased in the spectrum. In particular, peaks in all spectra at ~6800 are related to the water adsorbed on the surfaces. This peak at ~6800 is less distinct in commercial coatings due to their hydrophobic nature, whereas it is more prominent in TSIL coatings because the modification has provided a wettable surface. In addition, quaternary imidazolium salt coatings have shown greater CH and CH_2_ peaks in the first overtone within the range of 5600–6000, and these peaks are also more explicit in the second overtone at around 8300 due to the alkyl chain moieties on the molecular structure. In contrast, the alkyl constituent of commercial coatings is apparent in the first overtone region at ~5650–5800.

**Figure 4:**
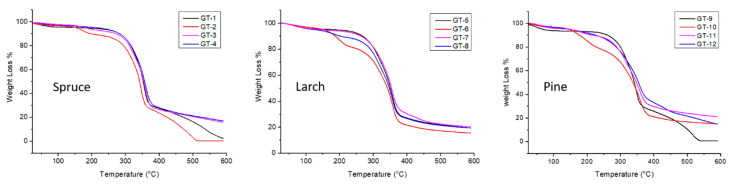
Thermograms of wood samples.

Further insight into the key features of surface coating concerning various kinds of wood was gained through thermogravimetric analysis (TGA), and the results are shown in Figure 4 and summarized in Table 1. In general, all materials have different responses to heat treatment due to their varied nature; therefore, this behavior regulates their suitability for practical applications. Surface treatment is expected to alter the thermal properties depending on the compatibility between the material and the coated layer. In this study, all materials except commercially coated ones exhibited one-step decomposition. All bare woods showed stable behavior until the temperature reached 200 °C with their first decomposition starting at ~240 °C. However, the first decomposition temperature decreased to ~150 °C after applying commercial coatings, as they formed an external layer made up of aromatic and hydrocarbon chains on the top of the wood, and it is typical for organic structures to decompose at this temperature. Moreover, Figure S4 shows the remaining final weight of each sample after the thermal process to evaluate its thermal durability. Concerning TSIL coatings, they exhibited the same trend as each piece of bare wood regarding the first decomposition temperature. Nonetheless, they demonstrated extended stability and slow weight loss during the increased temperature in spruce and pine, while there was a slight increase in the stability of larch after TSIL coatings were applied. This behavior can be attributed to the long hydrocarbon chains and sulfur–carbon (C-S) bond content of TSILs, which have intrinsic retardancy against flammability.

### Electrical Measurements

The use of wood floors in homes and activity centers, such as sports courts, is a popular choice due to the ease of installation, stiffness, and easy maintenance of wood-based components. Therefore, a wood-based TENG system is considered a sustainable and renewable way to harvest electrical energy from the mechanical motion of human activity. As with all renewable energy devices, it can be postulated that the surface coverage of wood is expected to influence charge formation and separation at the interface and improve the corresponding TENG device parameters.

**Figure 5:**
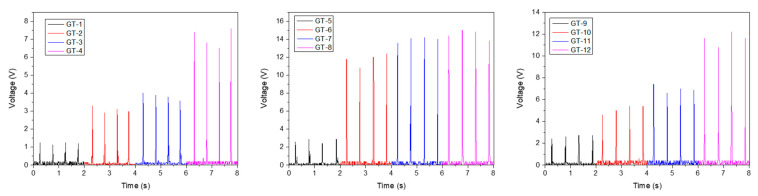
Instant output voltages of TENG devices with respect to various coatings.

The devices’ output performance was analyzed using vertical direction movement measurements obtained by manually striking them with a roughly constant 20 N force and 2 Hz frequency. Figure 5 depicts the instantaneous output voltages of the produced TENGs under the internal resistance of the used instrument. The maximum output voltages of the TENGs fabricated with bare woods were 1.24, 2.88, and 2.72 V for GT-1, GT-5, and GT-9, respectively. Among the bare woods, GT-5 exhibited the highest performance compared to the others. Furthermore, the surface coatings increased the instantaneous output voltages, with maximum measured values detected from the TSILs coating of 14.2 and 15.0 V for GT-7 and GT-8, respectively. It can be inferred that the imidazolium salt coating resulted in the rectification of voltage parameters due to their enhanced electron-donating ability, which also enhanced the tribopositive character of the wood samples.

**Figure 6:**
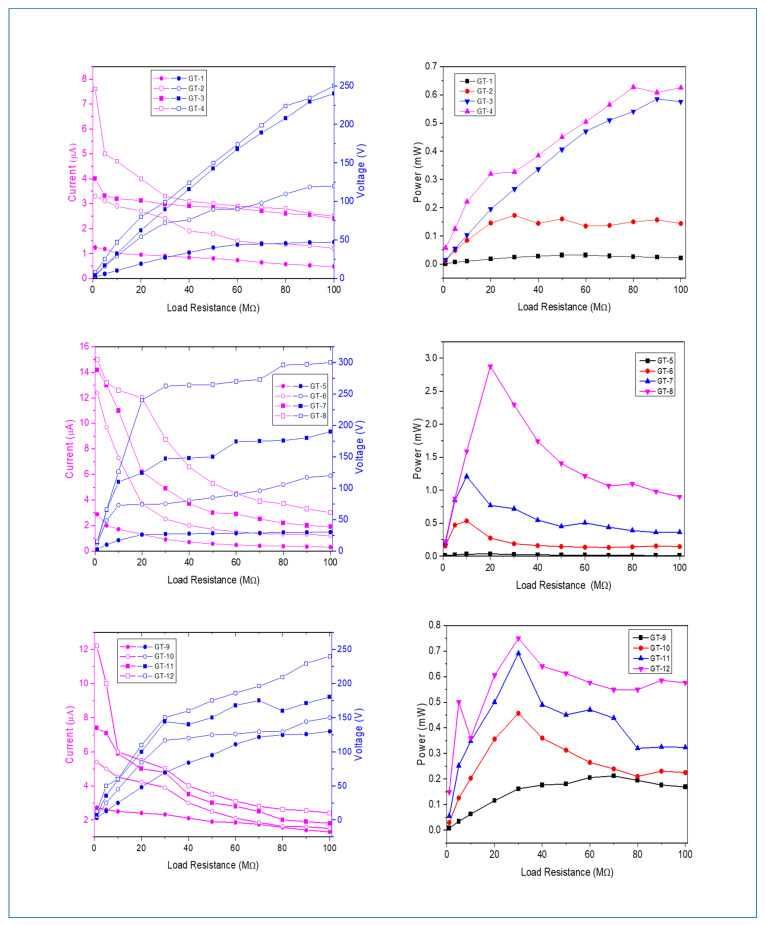
Current–voltage and maximum power curves of TENGs with different coated woods under varying loads.

Additional evaluation of the electrical performance was achieved by measuring the current–voltage changes of TENGs when altering loads, and the obtained results are illustrated in Figure 6. It is apparent from the graphs that the greater the load applied to the system, the lower the output current produced over time. Moreover, the increase in voltage continues until the highest stress point, owing to the minor influence of load increases up to this point. However, exceeding this point due to the rise in the applied load causes contrary effects on the current, and, consequently, the voltage starts to decrease. This distinctive variation is based on the nonlinear correlation between the applied load and measured power. Additionally, a comparison of the current–voltage results reveals that the surface coverage of wood samples exhibits improved electrical performance. The maximum measured voltage–current values are 300 V and 15.0 µA for GT-8 under maximum and minimum load conditions, respectively. The high performance of TSILs can be mainly attributed to ion pairing and ion aggregation, along with decreased ion mobility arising from their large ion sizes, which might enhance the tribopositive feature of the wood samples [28,61]. In the case of the commercial coating system, the slight improvement observed with respect to TSILs can be attributed to its aromatic ring and long hydrocarbon chain combination, which increases the electron density thanks to the formation of a more positive inductive effect [19,62].

The assessment of maximum power points (MPPs) depending on the various loads before and after surface modifications was carried out using the P = V^2^/R equation, and the results are shown in Figure 6. Since each sample has a different electrical response due to its diverse electrical behavior, each of them has shown its highest value at different loads. However, power curves follow similar variations with changing loads and approach 0 after exceeding MPP. In particular, TSIL-coated samples showed the best MPP values compared to the other samples, and GT-8 produced the highest MPP value of 2.88 mW at 20 MΩ. These results suggest that the surface grafting of wood samples is not only essential for the rectification of the electrical performance of the wood-based materials but also serves as an ideal modifier, similar to TSILs, and contributes to enhancing the charge generation, leading to an improvement in the output electric power through increased charge density at the contact surface of the TENGs. Moreover, the hydrophilic nature of TSILs allows for water–PTFE polarization when moisture in the air adheres to the surface that comes into contact with hydrophobic PTFE, further contributing to charge generation [57].

**Figure 7:**
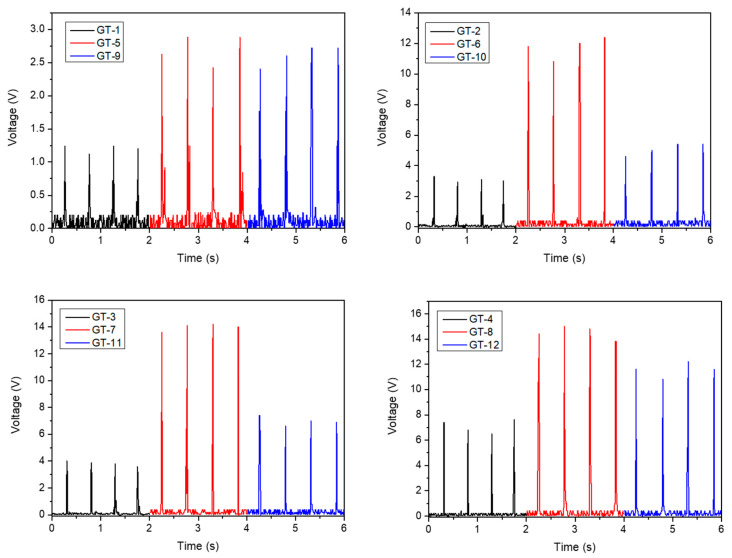
Comparison of wood samples in terms of instant output voltages in TENG devices.

**Figure 8:**
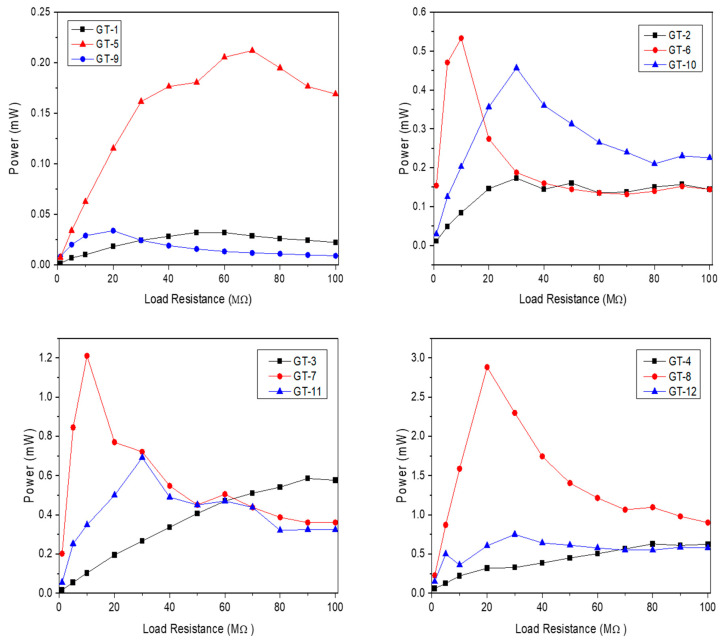
Comparison of wood samples in terms of maximum power points in TENG devices.

A set of calculations was performed to evaluate the electrical gain of TSILs relying on the surface coverage, and the results are illustrated in Figure 7 and Figure 8. As previously noted, GT-5 had the best instant voltage and MPP within the group of bare samples, which might indicate the highest tribopositive character of larch compared to spruce and pine. Concerning TSILs, salt with a shorter alkyl chain [C_6_-Im-CH_2_OMen][Sacc] performed better than salt with a longer alkyl chain [C_10_-Im-CH_2_OMen][Sacc] in all voltage and power trials. Consistent with the current result, our previous reports have described the role of the molecular size-dependent effectiveness of molecular packaging in the coverage of the surface and interface [63]. In general, the greater the molecular size, the greater the steric effect and, thereby, the less close packaging of bulky molecules [64]. Within this framework, it is also expected that treatment with salt that possesses a C6-carbon chain can bring about the highest settlement of the molecules throughout the cellular matrix of the wood structure. Hence, this could be a possible explanation for why [C_6_-Im-CH_2_OMen][Sacc] rectifies the electrical output better than [C_10_-Im-CH_2_OMen][Sacc].

## 3. Materials and Methods

### 3.1. Materials

The synthesis, purification, and detailed characterization of two selected TSILs, (i) 3-hexyl-1-[(1*R*,2*S*,5*R*)-(–)-menthoxymethyl]imidazolium saccharinate ([C_6_-Im-CH_2_Omen][Sacc]) and (ii) 3-decyl-1-[(1*R*,2*S*,5*R*)-(–)-menthoxymethyl]imidazolium saccharinate ([C_10_-Im-CH_2_Omen][Sacc]), were previously introduced to the literature by Kubis et al. [34]. Wood materials, namely larch, spruce, and pine, as well as a double-layered commercial coating system (Remmers HK Stain and Remmers Lasur UV as the bottom and top coats, respectively), were kindly provided by STAMADREW (https://www.stamadrew.pl/en, accessed on 20 September 2023, Czechowice-Dziedzice, Poland), the official distributor of certified SECA wood materials in Poland (Serafin Campestrini s.r.o. SECA Borohrádek, Czech Republic). Ethyl alcohol was purchased from Merck (Darmstadt, Germany).

### 3.2. Preparation of Wood Samples

Each “as received” wood sample was used without subjecting it to further processing before being cut into sample coupons. Each coupon was sliced to the following dimensions (h, l, w): 1 × 40 × 40 mm. Samples were prepared by slicing the wood into 1 × 40 × 40 mm^3^ pieces. The first coat of the commercial coating was applied to the surface with a brush, as described in the application manual, and cured for 48 h. The topcoat was applied by following the same procedure. As for the coating with TSILs, their excellent wood penetration abilities and their penetration depth have already been explicitly detailed in our previous report. In this follow-up study, each TSIL was dissolved in ethyl alcohol to prepare 0.5 mg/mL solutions. To control the consistency, an automatic pipet with 3 mL of solution was used to slowly dispense the same amount of solution on the distinct wood surface, and each sample was left to dry at room temperature overnight to be used in further stages. Following this procedure, 3 different TSIL-coated specimens were prepared from each used wood sample. All samples with acronyms, their surface treatments, and some physical properties are summarized in Table 1.

### 3.3. Device Fabrication

**Figure 9:**
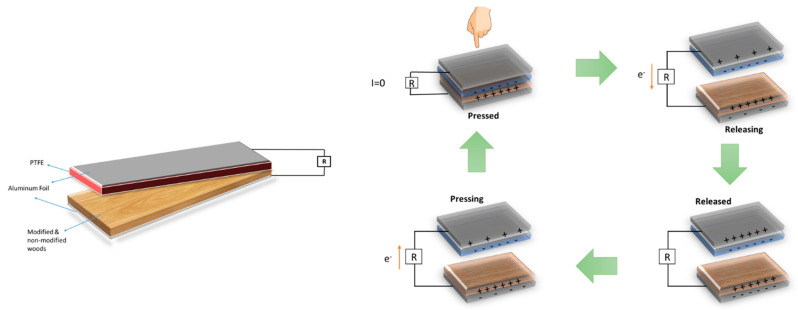
Layered structure of fabricated TENG devices and the working mechanism of TENG.

Figure 9 illustrates the structures of doped TSIL molecules (a), the TENG model used in this experiment (b), and the working principle of the device (c). The supporting layer of the designed devices is formed of acetate paper with dimensions of 1 (h) × 100 (l) × 100 (w) mm. Bare and surface-coated woods and Teflon tape samples were sized as 1 (h) × 40 (l) × 40 (w) mm and used, in turn, as positive and negative dielectrics. Opposite dielectric layers were glued to both sides of the acetate paper to avoid slippage from the supporting layer.

### 3.4. Instrumentation and Device Characterization

A Kruss DSA100E drop-shape analyzer (Germany) was used to determine the contact angle of the samples. The Tesla Bs 340 model SEM (Czech Republic) was used to obtain surface images of prepared wood sheets using the following parameters: EHT = 5.00kV, WD = 4.7–7.2 mm, and resolution = 100 μm. A Gw instek GDS-1152-U oscilloscope with a short-circuit resistance (Rin) of 1 MΩ was used to measure the voltage of the TENGs. Thermo Scientific Nicolet iS50 model NIR-IR spectroscopy (U.S.A.) device was used to analyze chemical bonds on the wood surfaces, and thermal characterizations of samples were carried out using Thermogravimetric TGA 550 TA Instruments (U.S.A.). In addition, power values were obtained by calculating equivalent resistance using the internal resistance of the oscilloscope. The electrical output was measured by converting the AC waveform of devices to a DC waveform through a bridge diode. Peak values of the electrical output of fabricated devices were regarded as references for voltage, current, and power curves.

## 4. Conclusions

Here, we evaluated the thermal and electrical performance of TSILs as surface-coating agents for wood samples to enhance their applicability in processes ranging from wood preservatives to smart floor systems. To assess the advantages and limitations of TSILs, a commercial coating system was used as a comparative material. We found that TSILs penetrated throughout the wood matrix, while commercial coatings formed an external layer on the surface. This is a favorable feature for materials engineering if further treatment is desired. Near-IR spectroscopy confirmed the presence of both TSILs and commercial coatings on wood surfaces with different spectral peaks. TGA analysis showed that all samples were thermally stable, but TSILs demonstrated extended thermal durability. Regarding electrical performance, TSILs outperformed bare and commercial coatings, with the highest result of 300 V and a ~5-fold increase in the maximum instant voltage value. The TSIL with a C6-carbon chain, [C_6_-Im-CH_2_OMen][Sacc], showed the highest rectification in electrical outputs among the TSILs, due to its smaller size. These findings provide insight into the potential use of TSILs as renewable and cost-efficient sources for smart floors to harvest energy from various vibrations in green construction.

## Figures and Tables

**Table 1:** Acronyms and some surface features of treated wood samples.

Sample	Wood	Coating	Contact Angle	Weight Loss
GT-1	Spruce	Bare	18.9°	97.6%
GT-2	Spruce	Commercial Coating	86.3°	99.7%
GT-3	Spruce	[C_10_-Im-CH_2_OMen][Sacc]	20.9°	84.1%
GT-4	Spruce	[C_6_-Im-CH_2_OMen][Sacc]	27.3°	83.1%
GT-5	Larch	Bare	32.05°	80.6%
GT-6	Larch	Commercial Coating	83.7°	84.5%
GT-7	Larch	[C_10_-Im-CH_2_OMen][Sacc]	22.2°	79.7%
GT-8	Larch	[C_6_-Im-CH_2_OMen][Sacc]	23.65°	80.6%
GT-9	Pine	Bare	34.45°	99.5%
GT-10	Pine	Commercial Coating	79.35°	85.0%
GT-11	Pine	[C_10_-Im-CH_2_OMen][Sacc]	22.2°	78.9%
GT-12	Pine	[C_6_-Im-CH_2_OMen][Sacc]	22.8°	85.2%

## Data Availability

The original data related to this research can be obtained at any time by contacting the corresponding authors via email: emre.arkan@us.edu.pl and miroslaw.chorazewski@us.edu.pl.

## Supplementary Materials

The following supporting information can be downloaded at: https://www.mdpi.com/article/10.3390/molecules28196758/s1, Figure S1: FT-IR spectra of modified and unmodified Spruce woods; Figure S2: FT-IR spectra of modified and unmodified Larch woods; Figure S3: FT-IR spectra of modified and unmodified Pine woods; Figure S4: Percentage of weight remaining of woods after TGA analysis.

## Sample Availability

Not available.
