# Supplementary material for: From Biological Source to Energy Harvesting Device: Surface Protective Ionic Liquid Coatings for Electrical Performance Enhancement of Wood-Based Electronics
Source: Molecules. 2023 Sep 22;28(19):6758. doi: 10.3390/molecules28196758 (PMC10574724; doi:10.3390/molecules28196758)
Supplement: Supplementary file 1 [file molecules-28-06758-s001.zip › molecules-2584822-supplementary.pdf]

Supplementary Material

From Biological Source to Energy Harvesting Device: Surface Protective Ionic Liquid Coatings for Electrical Performance Enhancement of Wood-Based Electronics

Gulnur Zharkenova[1,2], Emre Arkan[1]*, Mesude Zeliha Arkan[1], Joanna Feder-Kubis[3,4], Janusz Koperski[5], Turlybek Mussabayev[2], Mirosław Chorążewski[1,]*

[1]Institute of Chemistry, University of Silesia in Katowice, Szkolna 9, Katowice 40–006, Poland; flower_0525@mail.ru (G.Z.); mesude-zeliha.arkan@us.edu.pl (M.Z.A.)

[2]Department of Civil Engineering, L.N. Gumilyov Eurasian National University, Astana 010008, Kazakhstan; eti.enu@mail.ru

[3]Faculty of Chemistry, Wrocław University of Science and Technology, Wybrzeże Wyspiańskiego 27, Wrocław 50–370, Poland; joanna.feder-kubis@pwr.edu.pl

[4]Department of Inorganic Chemistry, Technische Universität Dresden, 01069 Dresden, Germany

[5]Institute of Physics, University of Silesia in Katowice, St 75 Pułku Piechoty 1, 41–500 Chorzów, Poland; janusz.koperski@us.edu.pl

*Correspondence: emre.arkan@us.edu.pl (E.A.), miroslaw.chorazewski@us.edu.pl (M.C.)

***Corresponding Authors:**

1-) Emre Arkan

e-mail: emre.arkan@us.edu.pl

ORCID ID: 0000-0002-9431-526X

2-) Mirosław Chorążewski

e-mail: miroslaw.chorazewski@us.edu.pl
ORCID ID: 0000-0002-8912-9024

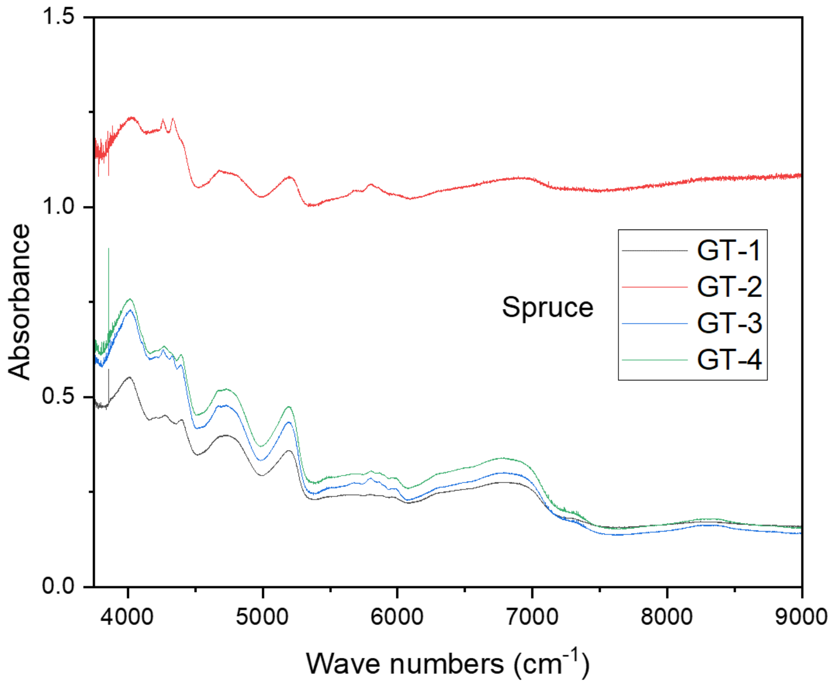

Figure S1: FT-IR spectra of modified and unmodified Spruce woods.

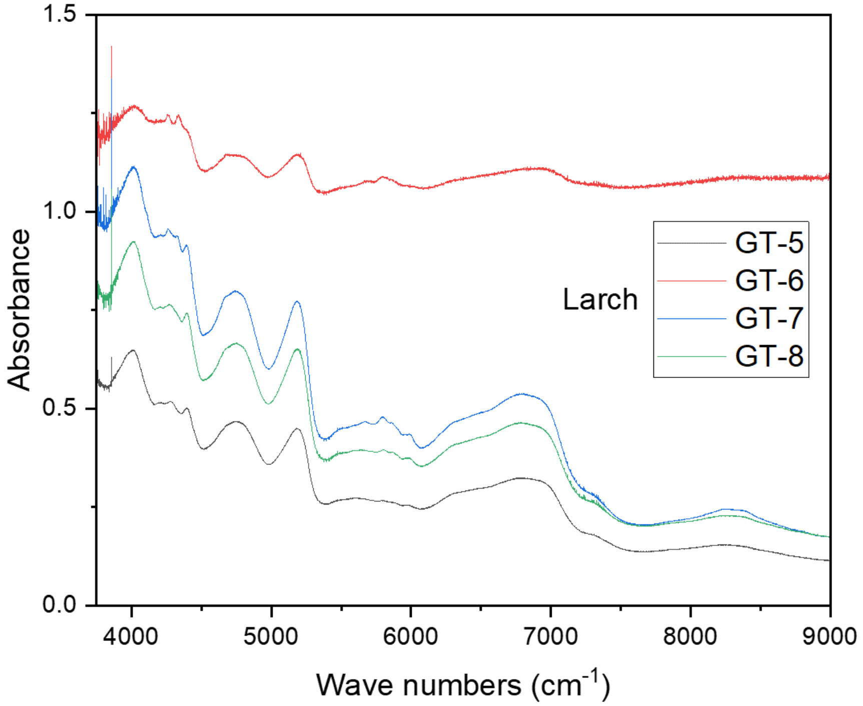

Figure S2: FT-IR spectra of modified and unmodified Larch woods.

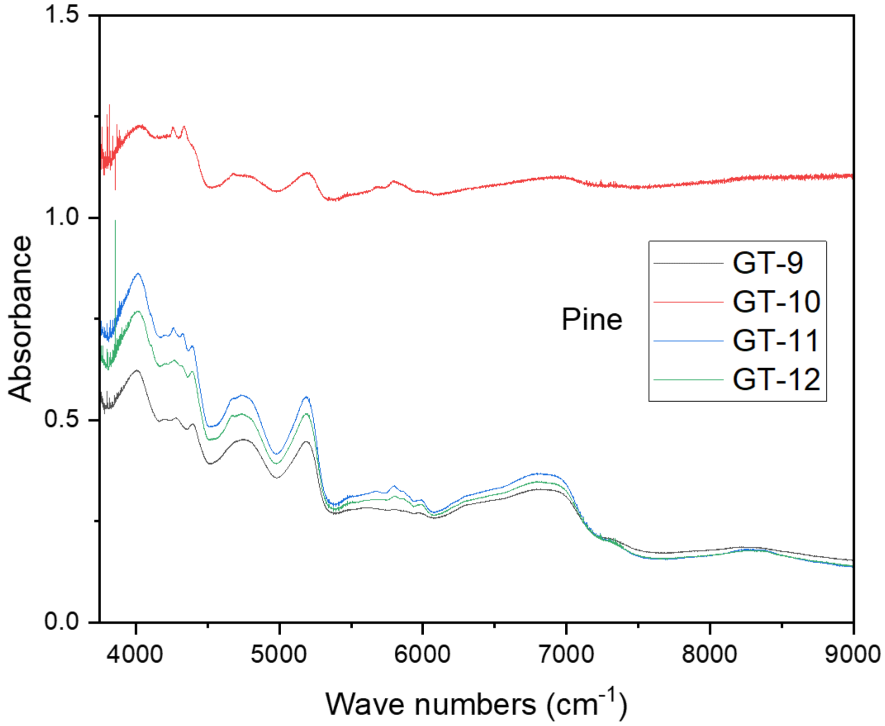

Figure S3: FT-IR spectra of modified and unmodified Pine woods.

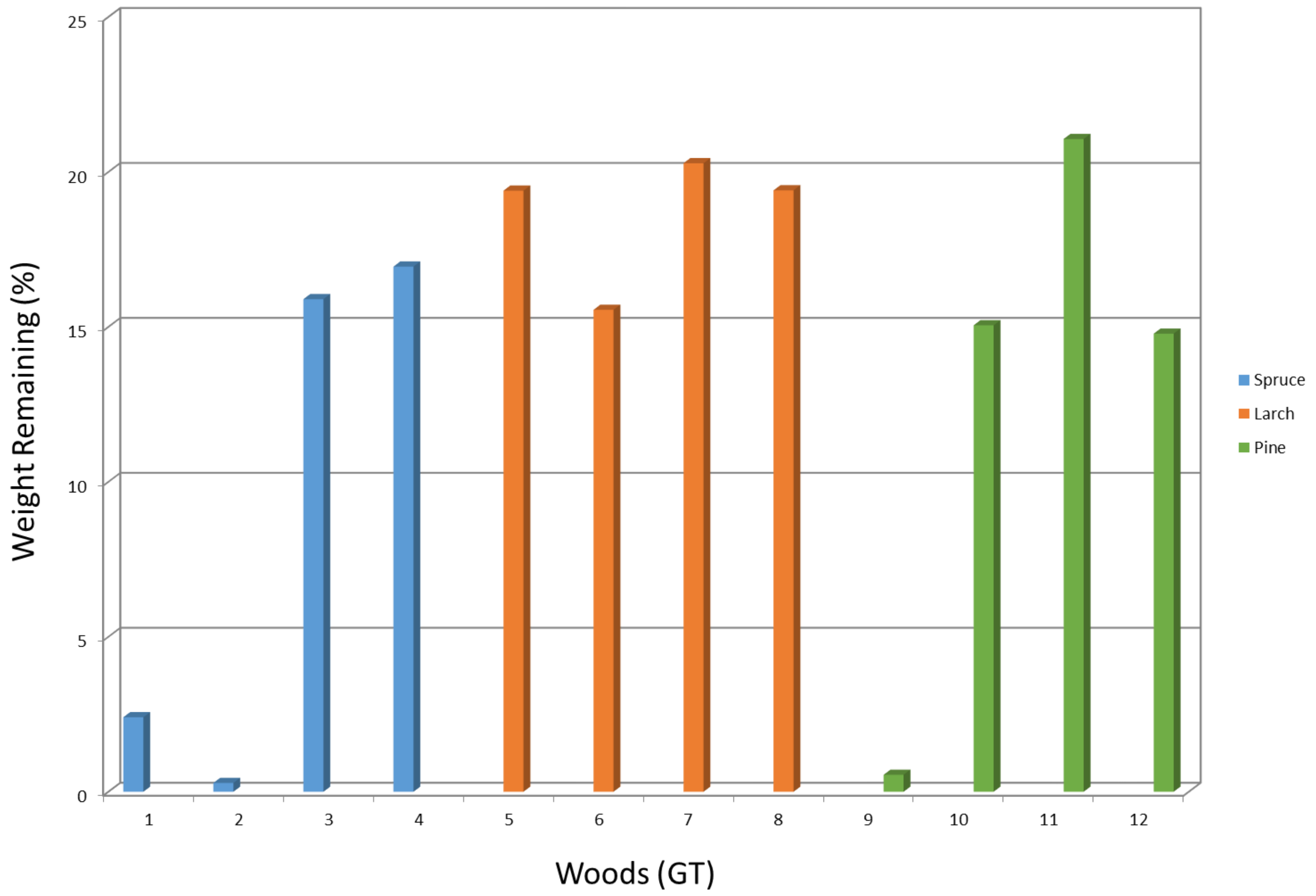

Figure S4: Percentage of weight remaining of woods after TGA analysis.
